# Genomic diversity of *Campylobacter jejuni* and *Campylobacter coli* isolated from the Ethiopian dairy supply chain

**DOI:** 10.1371/journal.pone.0305581

**Published:** 2024-08-19

**Authors:** Abera Admasie, Xiaoyuan Wei, Beth Johnson, Logan Burns, Preeti Pawar, Ashley Aurand-Cravens, Olena Voloshchuk, Edward G. Dudley, Tesfaye Sisay Tessema, Ashagrie Zewdu, Jasna Kovac

**Affiliations:** 1 Institute of Biotechnology, Addis Ababa University, Addis Ababa, Ethiopia; 2 Department of Biology, College of Natural and Computational Sciences, Arba Minch University, Arba Minch, Ethiopia; 3 Department of Food Science, The Pennsylvania State University, University Park, PA, United States of America; 4 Division of Laboratory Services, Kentucky Department of Public Health, Frankfort, KY, United States of America; 5 Center for Food Science and Nutrition, Addis Ababa University, Addis Ababa, Ethiopia; Universidad San Francisco de Quito, ECUADOR

## Abstract

Campylobacteriosis outbreaks have previously been linked to dairy foods. While the genetic diversity of *Campylobacter* is well understood in high-income countries, it is largely unknown in low-income countries, such as Ethiopia. This study therefore aimed to conduct the first genomic characterization of *Campylobacter* isolates from the Ethiopian dairy supply chain to aid in future epidemiological studies. Fourteen *C*. *jejuni* and four *C*. *coli* isolates were whole genome sequenced using an Illumina platform. Sequences were analyzed using the bioinformatics tools in the GalaxyTrakr platform to identify MLST types, and single nucleotide polymorphisms, and infer phylogenetic relationships among the studied isolates. Assembled genomes were further screened to detect antimicrobial resistance and virulence gene sequences. Among 14 *C*. *jejuni*, ST 2084 and ST 51, which belong to the clonal complexes ST-353 and ST-443, respectively, were identified. Among the 4 sequenced *C*. *coli* isolates, two isolates belonged to ST 1628 and two to ST 830 from the clonal complex ST-828. The isolates of *C*. *jejuni* ST 2084 and ST 51 carried β-lactam resistance gene *blaOXA-605*, a fluoroquinolone resistance-associated mutation T86I in the *gryA* gene, and a macrolide resistance-associated mutation A103V in *50S L22*. Only ST 2084 isolates carried the tetracycline resistance gene *tetO*. Conversely, all four *C*. *coli* ST 830 and ST 1628 isolates carried *tetO*, but only ST 1628 isolates also carried *blaOXA-605*. Lastly, *C*. *jejuni* ST 2084 isolates carried a total of 89 virulence genes, and ST 51 isolates carried up to 88 virulence genes. Among *C*. *coli*, ST 830 isolates carried 71 genes involved in virulence, whereas two ST 1628 isolates carried up to 82 genes involved in virulence. Isolates from all identified STs have previously been isolated from human clinical cases, demonstrating a potential food safety concern. This finding warrants further monitoring of *Campylobacter* in dairy foods in Ethiopia to better understand and manage the risks associated with *Campylobacter* contamination and transmission.

## Introduction

Campylobacteriosis is an infectious disease caused predominantly by *Campylobacter jejuni* and *Campylobacter coli* [1, 2]. It can be particularly severe in children under the age of five [3]. Following a *Campylobacter* infection, individuals may experience post-infection complications such as reactive arthritis, neurological disorders like Guillain-Barré syndrome, and chronic medical sequelae [4]. In the US, *Campylobacter* causes an estimated 1.5 million illnesses, 19,300 hospitalizations, and 240 fatalities annually [5]. EU nations reported 129,960 confirmed cases of campylobacteriosis in 2021 [6]. Studies have also shown that *Campylobacter* species are widespread among humans in sub-Saharan Africa, with a pooled prevalence of 9.9% [7]. In Ethiopia specifically, the prevalence of campylobacteriosis reported in published studies ranged from 8% to 20% [8–12].

*Campylobacter* is primarily transmitted through contaminated food, particularly raw or undercooked poultry meat, as well as unpasteurized milk and untreated water [13, 14]. Furthermore, outbreaks of campylobacteriosis have been linked to the consumption of contaminated dairy products [15]. For example, in 2016, public health departments in England reported 69 cases of campylobacteriosis linked to the consumption of raw milk sold at dairy farms [16]. Similarly, cases of campylobacteriosis were reported in Italy, where the consumption of raw milk resulted in an outbreak of *C*. *jejuni* [17]. Multiple outbreaks of campylobacteriosis have also been linked to the consumption of contaminated milk and cheese in the US [18–20]. However, less is known about foodborne campylobacteriosis and the prevalence of *Campylobacter* in dairy foods in Ethiopia.

The dairy value chain in Ethiopia is highly fragmented and includes approximately 500,000 smallholder rural farmers who produce approximately 1,130 million liters of milk. Milk is produced using various breeds of cattle, including indigenous breeds such as Boran, Arsi, and Horro, as well as crossbred cattle [21]. Crossbreeding programs have been implemented to improve the productivity of indigenous breeds, with the introduction of exotic breeds such as Holstein Friesian and Jersey [22]. The dairy value chain comprises traditional smallholder, privatized state, and urban and peri-urban farms. Smallholder rural farmers account for the majority of milk production, with 71% of the producers selling their milk directly to consumers. The sector also includes small-scale household farms in the highlands, which hold most of the potential for dairy development. The dairy value chain in Ethiopia is complex, with both formal and informal channels, and only 5% of the milk produced is sold in commercial markets [23]. The storage facilities in the Ethiopian dairy value chain are not well-developed, leading to challenges in milk collection, chilling, and preservation. There is a lack of infrastructure, including clean water, electricity, sanitation, roads, cooling plants, and refrigeration, making it difficult to maintain milk quality and safety [24]. The large number of smallholder rural farmers and the lack of well-coordinated feed production and distribution present challenges that negatively impact dairy production systems and the possibilities for the export of Ethiopian dairy products. As a result, the development of a vertically integrated and coordinated milk value chain is considered an important strategy for reduction of operational challenges [25].

In Ethiopia, there is a lack of data on foodborne outbreaks of campylobacteriosis. Furthermore, limited information is available on the prevalence of *Campylobacter* in the dairy supply chain. In our previous cross-sectional study in major Ethiopian milk sheds, we found that 11% of all tested dairy foods collected in a dry season were contaminated with *Campylobacter* [26]. Due to the lack of access to genotyping, it remains unknown whether *Campylobacter* isolates obtained from the Ethiopian dairy supply chain resemble those that are widespread globally or if they present unique genotypes endemic to Ethiopia.

Multi-locus sequence typing is a commonly used genotyping method that allows for the spatial and temporal comparison of the prevalence and distribution of *Campylobacter* genotypes [27]. The most prevalent MLST genotypes of *Campylobacter* reported in the global PubMLST database include sequence types (STs) 21, 45, and 50 [28]. While these genotypes are commonly found in high-income countries, they have not been reported in the limited published literature from sub-Saharan Africa [29]. To improve *Campylobacter* control strategies, a greater understanding of the prevalence and distribution of individual *Campylobacter* genotypes in the food systems in sub-Saharan Africa is needed. Isolate genotyping, including *in silico* MLST typing through whole-genome sequencing (WGS) can enhance source tracking and identification of major sources of human *Campylobacter* in the food systems, as well as support epidemiological investigation of outbreaks [30–32]. Beyond tracking the transmission of *Campylobacter* among environmental, agricultural, and human sources [33], WGS can also support the study of *Campylobacter* evolution and its antimicrobial resistance and genetic determinants contributing to virulence [34–36]. This study therefore employed whole-genome sequencing to characterize *C*. *jejuni* and *C*. *coli* isolated from the Ethiopian dairy supply and generate insight into the genetic diversity and genomic potential of isolates to cause human illness and resist antimicrobial treatments.

## Materials and methods

### Isolate collection and *Campylobacter* species confirmation

*Campylobacter* isolates that had previously been isolated from raw milk, pasteurized milk, and cottage cheese collected from Ethiopian regions of Amhara, Oromia, and the Southern Nations, Nationalities, and Peoples’ (SNNPR) between 2020 and 2021 were included in this study [26]. Isolates were cryopreserved in the brain heart infusion broth supplemented with 20% glycerol and maintained at -20°C. In 2022, 18 isolates that were successfully recovered were sent to the Pennsylvania State University for PCR confirmation [37] and then sent on to the laboratory of the Kentucky Department of Public Health for whole genome sequencing.

### DNA extraction and whole genome sequencing

DNA extraction was performed using the QIAcube Connect instrument with the DNeasy Blood and Tissue kit (Qiagen, Germantown, MD, USA). The extracted DNA was quantified using the Qubit 4 fluorometer (Thermo Fisher Scientific, Madison, WI, USA). Subsequently, library preparation was carried out using the Illumina DNA Prep kit (Illumina, San Diego, CA, USA). Nextera DNA CD indexes were used for sample indexing and indexed libraries were pooled in equimolar concentrations. Finally, the paired-end sequencing run was conducted on an Illumina MiSeq instrument, utilizing a v3 600-cycle kit (Illumina, San Diego, CA, USA).

### Genome assembly, quality control, annotation, multi-locus sequence typing, and taxonomic identification

The quality of raw sequencing reads was assessed using FastQC (Galaxy Version 3.0.3+galaxy0) [38], followed by trimming of low-quality bases and adapters with Trimmomatic by using default settings (Galaxy Version 0.39+galaxy0) [39]. The resulting trimmed sequences were assembled using Skesa (Galaxy Version 0.0.4) with default settings [40]. The genome assembly quality was assessed with Quast (Galaxy Version 5.2.0+galaxy1) [41]. SkesaMLST (Galaxy Version 0.0.4) was then applied to assemblies to determine multi-locus sequence types (MLST STs). GTDB-Tk (V2.1.0) with the reference database version R207_v2 was used for genome-based taxonomic identification [42]. The annotation of the 18 *Campylobacter* genomes was performed using Prokka (Galaxy Version 1.14.6+galaxy0) [43], and Roary (Galaxy Version 3.13.0+galaxy2) was used to calculate the pan-genome. The output of Roary was visualized using a roary plot (Galaxy Version 1.0) [44].

### Single nucleotide polymorphism detection and phylogenetic analysis

High-quality single nucleotide polymorphisms (SNPs) were detected using the CFSAN SNP pipeline on the GalaxyTrakr platform [45, 46]. For SNP analyses of *C*. *jejuni* isolates, we used a reference genome of *C*. *jejuni* SRR24187546, and for SNP analyses of *C*. *coli* the reference genome of *C*. *coli* SRR24187539. The reference genomes were selected based on the high quality of genome assembly, as determined using the N50 metric. The identified SNPs were used to construct a maximum likelihood phylogenetic tree using IQ-TREE (Galaxy Version 2.1.2+galaxy2) with default settings and 1,000 rapid bootstrap iterations [47].

### Identification of antimicrobial resistance and virulence genes

ABRicate (Galaxy Version 1.0.1) [48] was used with default settings to detect the presence of virulence factor and antimicrobial resistance gene sequences in the studied isolates by utilizing the Virulence Factor Database (VFDB) and the NCBI Bacterial Resistance Reference Gene Database, respectively. Amrfinderplus_db NCBI (Galaxy Version 3.11.11+ galaxy1) was used to detect resistance mutations T86I in *gyrA* and A103V in *50S_L22* [49].

### Comparison of genomes of the studied isolates with those available in the Pathogen Detection database

Isolates studied here were submitted to the NCBI Pathogen Detection database to compare their genomic similarity with the *Campylobacter* isolates available in the database. Specifically, we identified the minimum SNP distance between our isolates and *Campylobacter* isolates of the same (non-human) source and between our isolates and *Campylobacter* isolates from a human source.

### Biofilm formation

Isolates were grown on mCCDA for 24 h at 42°C in microaerobic conditions (5% O_2_, 10% CO_2_, in N_2_). Subsequently, cultures were grown in BHI broth for 24 h at the same conditions, and then adjusted to OD_600_ of 0.2. Adjusted cultures were loaded into minimal biofilm eradication concentration (MBEC) plates in 8 replicates and allowed to form biofilm for 72 h at 42°C in microaerobic conditions. Each plate included 8 negative controls (just BHI) and 8 wells filled with a control strain, *C*. *jejuni* ATCC 33560. After completed incubation, biofilm formation on pegs was quantified using the crystal violet assay [50] as follows: biofilms were rinsed for 10 s by submerging pegs in sterile 0.85% NaCl, followed by heat-fixation at 65°C for 45 min in an air incubator. Subsequently, pegs were submerged in 1% w/v of crystal violet solution (Ward’s Science, Rochester, NY) and incubated at room temperature for 45 min. After staining, pegs were rinsed by two 10 s submersions in fresh sterile 0.85% NaCl, and de-colorized with 95% ethanol for 15 min. Lastly, the absorbance of crystal violet-ethanol solution was measured at 570 nm using a BioTek Synergy Neo2 microplate reader (Thermo Fisher Scientific, Waltman, NJ). The experiment was repeated in three biological replicates. The data were plotted as averages with standard deviations for each isolate.

### Statistical analysis

The association between the known genetic determinants of antimicrobial resistance and phenotypic antimicrobial resistance published in our previous study [116] was assessed by calculating the sensitivity and specificity. The statistical significance of differences in isolates’ biofilm formation compared to the negative control was assessed using a t-test in RStudio 4.3.3 [51].

## Results

### Four MLST sequence types were detected among the studied isolates

The median genome assembly size of the 14 *C*. *jejuni* isolates was 1.76 Mbp, the median N50 was 0.16 Mbp, and the median GC% content was 30.3%. Among the four *C*. *coli* genomes, the median genome assembly size, GC%, and the median N50 were 1.69 Mbp, 31%, and 0.18 Mbp, respectively. The isolate metadata and genome quality information are reported in the S1 Table in S1 File. The pangenome analysis was conducted for just *C*. *jejuni* isolates, as there were too few *C*. *coli* isolates available in our dataset. Among the 14 *C*. *jejuni* genomes, a total of 3,412 genes were detected, of which 309 were core genes detected in all studied isolates (S2 Table in S1 File). No softcore genes, a total of 2,822 shell genes, and 284 cloud genes were detected. Shell genes represented approximately 82% of the total gene count.

**Fig 1:**
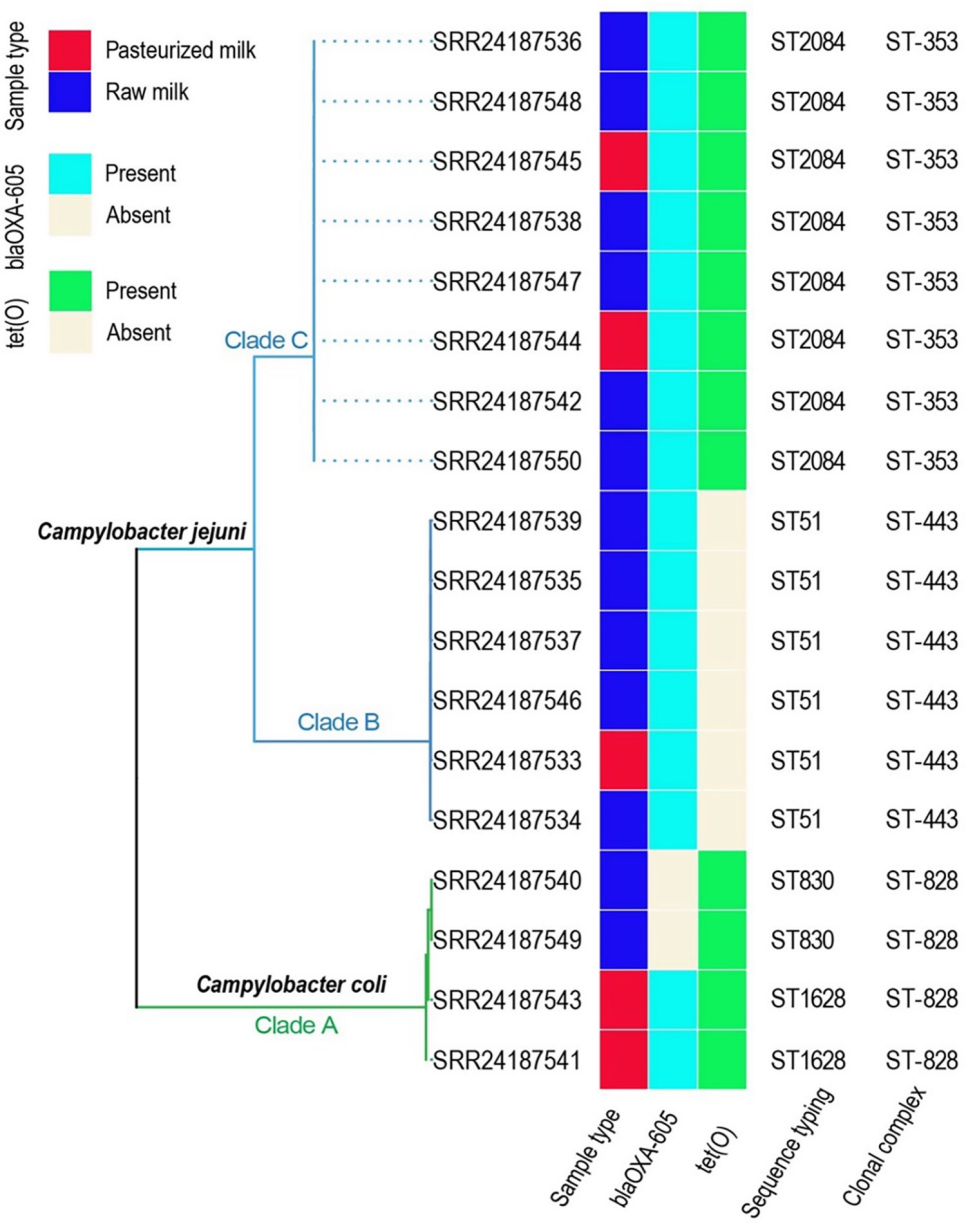
Phylogenetic tree constructed based on the high-quality single nucleotide polymorphisms (SNPs) identified using the FDA CFSAN pipeline among the 14 *Campylobacter jejuni* and 4 *Campylobacter coli* isolates obtained from dairy foods in Ethiopia.

Further, a total of 2 sequence types and 2 clonal complexes were identified among *C*. *jejuni* isolates. Specifically, ST 2084, which belongs to the clonal complex ST-353, was the predominant sequence type. The remaining six isolates belonged to ST 51, which is part of the clonal complex ST-443. Isolates of *C*. *coli* clustered in clade A, separately from isolates of *C*. *jejuni* in the constructed maximum likelihood tree (Fig 1). Specifically, isolates of *C*. *jejuni* clustered in two distinct clades (B and C) in the constructed maximum likelihood tree (Fig 1). Clade B included 6 isolates of ST 51, while clade C was comprised of 8 isolates of ST 2084. Notably, ST 51 and 2084 were detected in both raw and pasteurized milk samples. Isolates within clade B and clade C differed by 1–5 and 0 SNPs, respectively (S3 Table in S1 File). Among *C*. *coli* isolates, we found two sequence types, ST 1628 and ST 830, which belonged to a single clonal complex, ST-828 (Fig 1). Isolates within clade A differed by 0–12 SNPs. *C*. *coli* ST 1628 was obtained from pasteurized milk, while ST 830 was obtained from raw milk.

### The majority of *C*. *jejuni* isolates carried a fluoroquinolone resistance mutation T86I in the *gryA* gene

In addition to assessing genomic similarity, we screened genomes for antimicrobial resistance gene sequences. Overall, we detected two antimicrobial resistance genes among studied *Campylobacter* isolates (Fig 1, S4 Table in S1 File). All *C*. *jejuni* isolates carried a *blaOXA-605* gene, but only ST 2084 isolates carried the *tetO* gene. Conversely, among *C*. *coli*, both ST 830 and ST 1628 isolates carried *tetO*, but only ST 1628 also carried the *blaOXA-605* gene. Based on the phenotypic antimicrobial resistance published in our previous study [26], the presence of gene *tetO* predicted tetracycline resistance with 80% sensitivity and 100% specificity. Furthermore, ten isolates of *C*. *jejuni* ST 2084 and ST 1628 had a T86I mutation in the *gryA* gene, which has been previously associated with resistance to quinolone antibiotics, such as ciprofloxacin and nalidixic acid. However, the resistance mutation in *gryA* predicted phenotypic resistance to ciprofloxacin with just 60% sensitivity and 67% specificity [26]. All *C*. *jejuni* isolates carried the A103V mutation in the 50S L22 mutation, which has previously been associated with the resistance of macrolide antibiotics, such as erythromycin. The phenotypic resistance to erythromycin was predicted with 92% sensitivity and 50% specificity based on the presence of a mutation in 50S L22 [26]. Lastly, in terms of heavy metal resistance genes, the arsenic resistance gene *arsP* was found in eight *C*. *jejuni* ST 2084 isolates and no other studied isolates (S5 Table in S1 File).

### All *C*. *jejuni* isolates carried genes encoding cytolethal distending toxin

**Fig 2:**
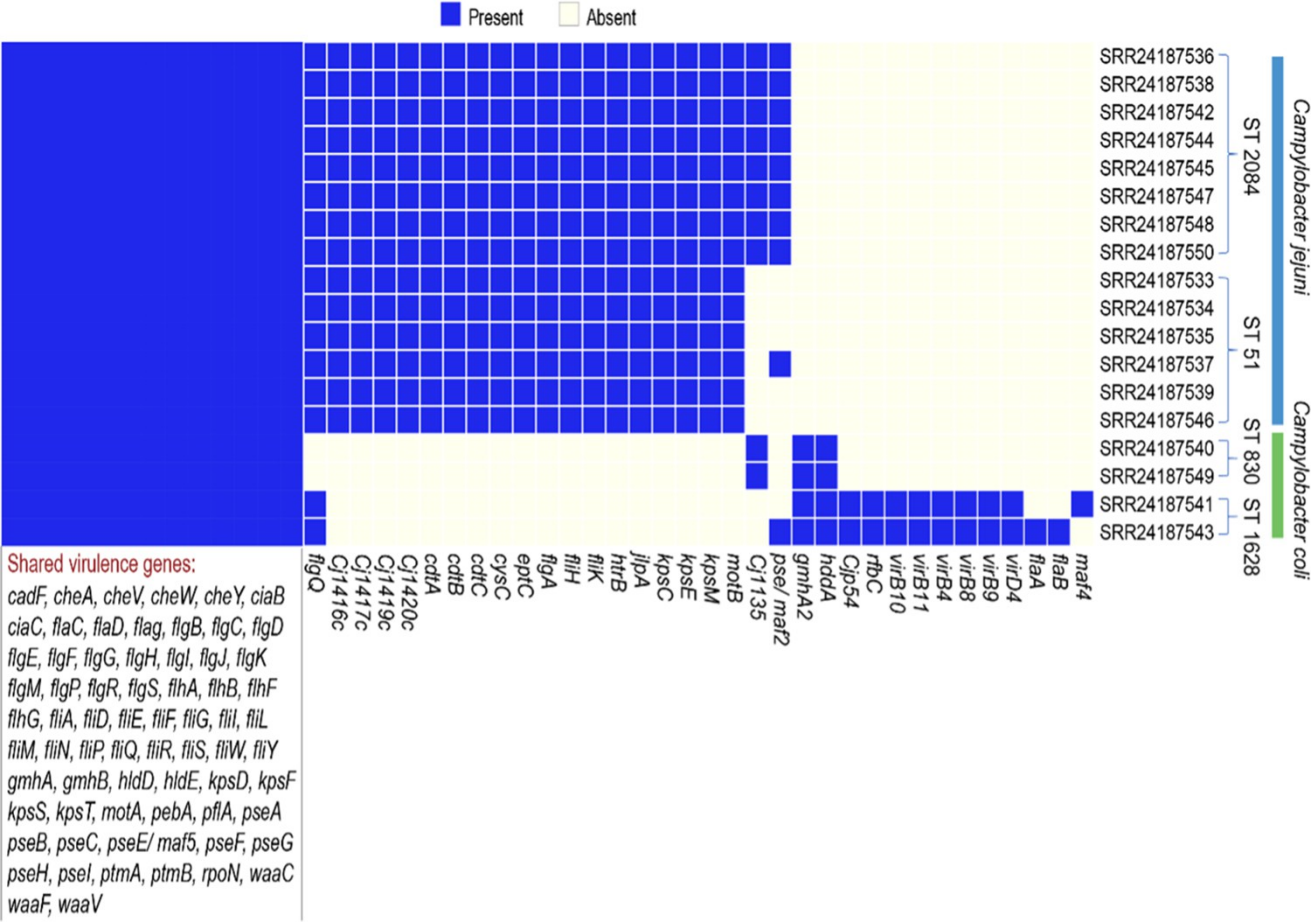
Virulence gene profiles detected in *Campylobacter jejuni* and *Campylobacter coli* isolates. The presence of a gene is indicated in blue and the absence is denoted in a light yellow color.

In this study, all *Campylobacter* isolates carried genes contributing to virulence, including *cadF*, *cheAVWY*, *ciaBC*, *flaCDG*, *flgBCDEFHIJKMPRS*, *flhABFG*, *fliADEFGILMNPQRSWY*, *gmhAB*, *hldDE*, *kpsDFST*, *motA*, *pebA*, *pflA*, *pseABCEFGHI*, *ptmAB*, *rpoN*, *waaCFV* (Fig 2). Furthermore, all *C*. *jejuni* isolates carried virulence genes *flgQ*, *Cj1416c*, *cj1417c*, *cj1419c*, *cj1420c*, *ctdABC*, *cysC*, *eptC*, *flgA*, *fliHK*, *htrB*, *jlpA*, *kpsECM* and *motB*, with ST 2084 harboring two additional genes, *Cj1135* and *pseD/maf2*, compared to ST 51. All *C*. *jejuni* isolates carried the *cdtABC* gene cluster encoding cytolethal distending toxin (Fig 2). One isolate from ST 51 also carried the *pseD/maf2* gene, which encodes the motility accessory factor PseD. The genes *gmhA2* and *hddA* were detected in all studied *C*. *coli* isolates. Among isolates of ST 830, we detected *cj1135*, which encodes a lipo-oligosaccharide. The ST 1628 *C*. *coli* isolates shared the virulence genes *virB8-11*, *virB4*, and *virD4*, and one isolate from ST 1628 carried additional virulence genes *flaAB* and *pseD/maf2*. A list of the detected genes and the corresponding products is available in the S6 Table in S1 File.

### *Campylobacter* isolates from Ethiopia were distinct compared to isolates from other geographical origins

Lastly, utilizing the Pathogen Detection database, we compared the 14 *C*. *jejuni* and 4 *C*. *coli* from this study with isolates from non-human (i.e., environmental, food, animal), and human sources that were available in the database. Our isolates clustered into four Pathogen Detection clusters and none of these four clusters contained isolates from other studies or countries, indicating distinct genetic profiles of isolates characterized in this study. The *Campylobacter coli* isolates from raw milk and pasteurized milk (SRR24187543 - PS02412 and SRR24187541 - PS02436) clustered in the Pathogen Detection SNP cluster PDS000145444.1. *C*. *coli* isolated from raw milk (SRR24187540 - PS02419 and SRR24187549 - PS02411) clustered in the Pathogen Detection SNP cluster PDS000145445.1. Additionally, the *C*. *jejuni* isolates from pasteurized and raw milk clustered in the SNP cluster PDS000145443.1 (SRR24187538 - PS02422, SRR24187550 - PS02404, SRR24187536 - PS02424, SRR24187548 - PS02439, SRR24187547 - PS02440, SRR24187545 - PS02432, SRR24187544 - PS02435, SRR24187542 - PS02418) and SNP cluster PDS000145442.1 (SRR24187539 - PS02421, SRR24187537 - PS02423, SRR24187535 - PS02425, SRR24187534 - PS02426, SRR24187533 - PS02430, and SRR24187546 - PS2441). Noteworthy, isolates from pasteurized and raw milk were found in the same clusters.

### Biofilm formation in *Campylobacter coli* and *Campylobacter jejuni*

**Fig 3:**
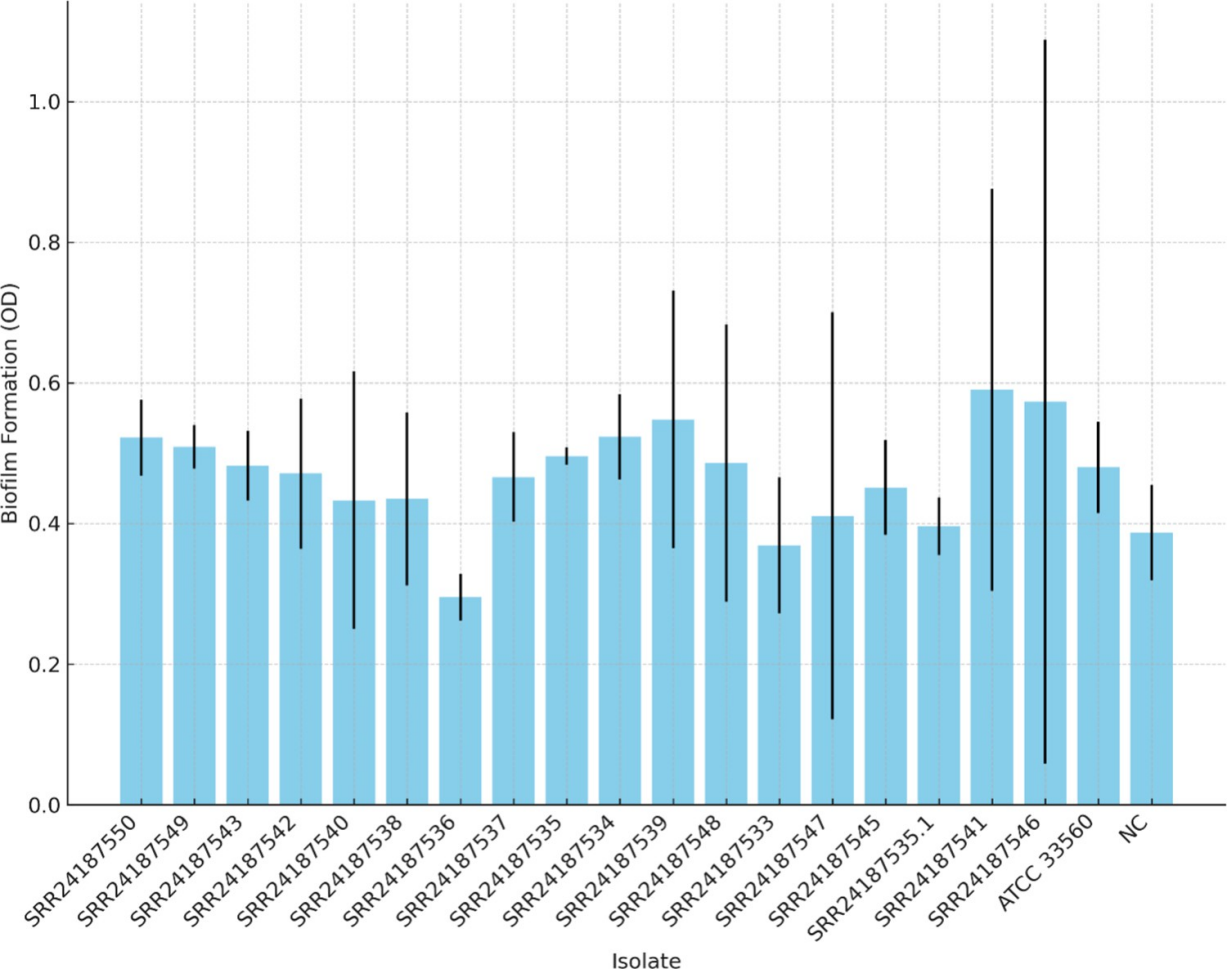
Biofilm formation by studied *Campylobacter jejuni* and *Campylobacter coli* isolates.

As shown in the Fig 3, none of the isolates formed significantly more biofilm compared to the negative control. Furthermore, we did not find significant difference in biofilm forming ability among tested isolates.

## Discussion

### MLST sequence types 2084 and 51 were detected in both raw and pasteurized milk

Among 14 *C*. *jejuni*, STs 2084 and 51, with ST-353 and ST-443 as their respective clonal complexes were identified in both pasteurized and raw milk. There is limited information available on the global prevalence of *Campylobacter jejuni* ST-2084. Based on the PubMLST database records, it is evident that ST 2084 isolates have previously been detected in the USA, UK, and Pakistan (S7 Table in S1 File) [52], however, a literature search did not yield any published papers reporting this particular sequence type [53]. Nevertheless, the clonal complexes to which these two STs belong have previously been reported in studies involving both humans and chickens. For example, Kinana et al. reported that the ST-353 complex was the most common complex detected among isolates from chicken carcasses in Senegal [54]. Manning et al. identified the ST-353 complex isolate in poultry in the UK. In China, Estonia, and Poland, ST-353 *C*. *jejuni* was isolated from retail chicken, humans, and chicken [55–57]. Other research has linked ST-353 isolates to human disease [58]. This included a study conducted in Michigan, US, in which they found ST-353 to be the third most abundant (11.8%) clonal complex among the 214 strains of *C*. *jejuni* recovered from patients with gastroenteritis [59].

Isolates belonging to ST-443 have previously been detected in Iran, Poland, China, as well as in the Gambia [55, 56, 60]. Among clonal complex ST-443 isolates, the ST 51 was found in human feces in Poland, Germany, and Croatia, and ranked among the top 10 STs detected across Europe [61–63]. ST 51 isolates were also isolated from patients undergoing treatment at the Red Cross War Memorial Children’s Hospital in Cape Town, South Africa [29]. Unlike our study, others have not previously reported isolating ST 51 from dairy food sources.

We detected *C*. *coli* ST 830 in raw milk and ST 1628 in pasteurized milk. Both of these sequence types belong to the clonal complex ST-828. While ST 830 has been detected in chicken meat in the Middle East, Asia, South America, and the US, the only record of its isolation in Africa (Egypt and Nigeria) from cattle and human feces exists in the PubMLST database (S2 Table in S1 File) [52, 64, 65]. Regarding ST 1628, before our study, there were no documented reports of this ST in Africa. However, the PubMLST database records suggest its detection in Europe, Asia, and North America (S2 Table in S1 File) from various sources, including cattle, chicken, beef, and chicken meat, environmental water, goose, and human stool [52, 66, 67].

The detection of the same STs in both raw and pasteurized milk in our study raises contamination concerns, suggesting that *C*. *jejuni* contamination from raw milk persists in pasteurized milk through processing, which could be due to pasteurization not being conducted at the appropriate temperature and for an appropriate time. The presence of the same genotypes in raw and pasteurized milk suggests a common contamination source, which could be contaminated water, equipment, or processing facility environment [68, 69]. Identifying and addressing the source of contamination is crucial for mitigating further contamination [70]. Moreover, the presence of *C*. *jejuni* in pasteurized milk underscores the possibility of two critical issues. First, it raises concerns regarding the effectiveness of the pasteurization process, suggesting that it may not have achieved the required temperature and duration necessary to eliminate pathogenic bacteria like *C*. *jejuni* [71–73]. Second, it highlights the potential for post-processing contamination, which could occur during subsequent stages such as milk handling, filling, or packaging [74]. Previous studies conducted in Norway and Sweden have uncovered instances of spoilage bacteria recontamination during the pasteurized milk filling process [75, 76]. Further, previous research found that the detection of *Escherichia coli* in pasteurized milk may be a result of pasteurization process failures or contamination during post-pasteurization processing [77]. This is particularly concerning because pasteurized milk is expected to be pathogen-free due to the rigorous heat treatment it undergoes.

### All studied *Campylobacter jejuni* isolates carried the genes encoding the cytolethal distending toxin

We have identified several virulence genes that are crucial for *Campylobacter* pathogenesis. All *Campylobacter* isolates from milk and milk products carried virulence gene involved in motility and flagellar biosynthesis (*motA*, *flaDG*, *flgABCDEFGHIJKMPQRS*, *flhABFG*, *fliADEFGHIKLMNPQRSWY*, *pseABC*, and *rpoN)*. The motility genes *motB*, *flaAB*, and *flgQ* were detected in all *C*. *jejuni*, *C*. *coli* PS02412 and all STs except ST 830 (PS02421 and PS02422), respectively. Genes *flgABCDEFGHIJKMPQRS* are involved in the assembly and structure of the flagellar rod, hook, and basal body [78], and genes *fliPQRSWY* contribute to the construction, control, and operation of the flagellar motor and filament [79–81]. The *motAB* encodes a stator complex protein that facilitates torque generation in the flagellar motor [82]. Moreover, motility regulation genes that were detected included *rpoN*, the product of which promotes the transcription of rod and hook components and the minor flagellin *flaB* [83], and *flhFG* genes that regulate the expression of the flagellar genes and the assembly of the flagellar basal body [84, 85]. The flagellar T3SS is located within the flagellum of *Campylobacter* [86]. T3SS secretion system genes (*flhAB*, *fliPQR*) encode the export gate proteins and play important roles in the regulation and assembly of the flagellar type III secretion system in *Campylobacter* [87–91]. This system is involved in the secretion of various proteins, including the Cia proteins, Fed proteins, and flagellin C [92]. Additionally, the type IV secretion system-related genes (*virB4*, *virB8-11*, and *virD4*) were only found in the genome of *C*. *coli* ST 1628. In a study conducted in Peru and Chile, T4SS genes like *virB4*, *virB9-11*, and *virD4* were found in *C*. *jejuni* and *C*. *coli* [93, 94].

*Campylobacter jejuni* uses flagella and chemotaxis to navigate its motility towards or away from environmental stimuli and they plays an essential role in pathogenesis during host colonization [95]. The flagellar assembly and the chemotactic motility depend on the expression of chemotaxis genes (*cheAYV*, or *cheW*) [96]. In this study, the chemotaxis-related genes (*cheAYV*) were detected in all *C*. *jejuni* and *C*. *coli* genomes.

Adhesion is a critical stage in pathogenesis before invasion and the release of toxins [97]. The adhesins of *Campylobacter* species facilitate a specific interaction between the bacterium and host cells [98]. In our study, the genes encoding adhesion-related proteins were found in all *Campylobacter jejuni* and *Campylobacter coli*. These included outer membrane proteins (OMPs) genes (*cadF*, *pebA*, *JlpA*), lipooligosaccharides (LOS) genes (*gmhA2BA*, *rfbC*, *hldDE*, *hddA*, *waaFCV*, and *htrB)*, lipopolysaccharides (LPS) genes (*KpsCSDEMT*, *Cj1416c*-*Cj1420c* and *cysC)*. This finding is consistent with other studies [99–101]. The genes *waaF*, *waaC*, and *waaV* have been associated with the biosynthesis of lipooligosaccharides (LOS) in *Campylobacter jejuni*. LOS structures have been implicated in the pathogenesis of Guillain-Barré Syndrome (GBS) due to molecular mimicry between LOS and human gangliosides, which can lead to the development of GBS. The association between LOS structures, including those influenced by *waaF*, *waaC*, and *waaV*, and the development of GBS is an active area of research, and further studies are needed to fully elucidate the role of these genes in GBS pathogenesis [102, 103].

All of our studied *Campylobacter* isolates carried *ciaBC* genes that code for Cia proteins, which are essential for invasion and colonization [87, 104, 105]. All of our studied *Campylobacte*r isolates also carried the *flaC* gene that encodes the FlaC protein that influences cell invasion by binding to epithelial cells [105]. The Cia proteins induce pathogen uptake and perhaps alterations in cellular responses only if delivered into the cytosol of the host target cell via bacteria-host cell contact [106].

In addition to adhesion and invasion, the ability of *Campylobacter* spp. to produce the cytolethal-distending toxin (CDT) is a crucial component of their pathogenicity. All *C*. *jejuni* studied here carried the *cdtABC* genes encoding for CDT. This toxin causes direct DNA damage leading to the invocation of DNA damage responses in human cells and leading to cell apoptosis [107]. The *cdt* gene cluster has been commonly detected in *Campylobacter* species isolated from humans [108], poultry [109], cattle, and swine isolates and contributes to campylobacteriosis [110, 111]. Overall, the presence of multiple important virulence factor gene sequences in the genomes of the studied *Campylobacter* isolates suggests their potential to cause foodborne illness.

We found that isolates studied here clustered into four pathogen detection clusters, but none of these four clusters contained isolates from other studies or countries, indicating distinct genetic profiles of isolates characterized in this study. The probable reasons for such isolation could be unique local environmental factors, specific transmission dynamics within the studied region, or the presence of distinct genetic lineages in the local pathogen population. The distinct genetic diversity of *Campylobacter* in Ethiopia compared to other countries could be influenced by a combination of environmental, antimicrobial, methodological, and epidemiological factors. Further research is needed to fully elucidate the reasons behind this distinct genetic diversity.

### The majority of isolates carried a resistance mutation in *gryA*

Antimicrobial resistance has been emerging among *Campylobacter*, which poses a serious risk for failed antimicrobial treatment of campylobacteriosis [13, 112]. Concerns about public health have arisen specifically due to the rising prevalence of fluoroquinolone-resistant *Campylobacter* [113, 114]. A study based in Oxford, UK, indicated that in 1995, 7% of the overall *Campylobacter* isolates were resistant to fluoroquinolones and by 2008 the prevalence had risen by nearly 2 percent [115]. We detected resistance mutations in *gryA* and the gene encoding 50S L22 in 55% and 78% of the isolates examined, respectively. This finding is consistent with our earlier research, which found that 57% of tested *Campylobacte*r isolates were phenotypically resistant to ciprofloxacin and erythromycin [116]. Ciprofloxacin resistance in *C*. *jejuni* is mediated by mutations in the *gyrA* gene, with the point mutation T86I being the most common one [117, 118], as reported elsewhere, including in Portugal, Botswana, and Nigeria [119–121]. Among *Campylobacter* isolated from human and poultry meat sources in Pennsylvania, USA, there was a strong correlation found between the specific resistance genetic determinants and the phenotypic resistance of ciprofloxacin [122].

Remarkably, in all *Campylobacter jejuni* isolates tested here, an A103V mutation in the gene encoding 50S L22 was detected. The 50S macrolide-binding site is composed of portions of the 23S rRNA subunit, ribosomal protein L4, and ribosomal protein L22 [123, 124]. The 50S L22 protein is a component of the 50S ribosomal subunit, and alterations in this protein have been associated with macrolide resistance in *Campylobacter* species [123, 124]. Another study identified the point mutation in 50S L22 as a resistance mechanism in *C*. *coli* isolates [125]. These point mutations are causing the rise of macrolide-resistant *Campylobacte*r strains, and consequently, macrolides such as erythromycin and azithromycin are becoming less effective in treating *Campylobacter* infections [125].

The genomic analysis revealed that 67% (12/18) and 89% (16/18) of studied *Campylobacter* carried *tetO* and *OXA-605*, which confer tetracycline and beta-lactam resistance, respectively. In our previous study, we reported that 89% of *Campylobacter* isolates, including isolates characterized here, were phenotypically resistant to tetracycline [116]. Here, we found that *tetO* gene was a highly sensitive and specific predictor of phenotypic resistance to tetracycline in studied *Campylobacter* isolates. Similarly, in studies from Korea and Peru, all tested *Campylobacter* isolates obtained from chicken carried the *tetO* gene [126, 127]. A study conducted in South Africa also found that the *tetO* gene was the most prevalent antimicrobial resistance gene detected in *Campylobacter* isolates from chickens and humans, with a prevalence of 68% and 64%, respectively [128]. Antibiotic resistance due to the production of D-lactamase OXA-61 was previously reported by Alfredson and Korolik [129]. Additionally, a UK investigation revealed that the isolates from both humans and poultry included OXA-61, which codes for the generation of β-lactamase and causes ampicillin resistance [130]. The function of this gene was confirmed with the insertional inactivation of blaOXA-61 which increased the susceptibility of *Campylobacter* to ampicillin, co-amoxiclav, penicillin, carbenicillin, oxacillin, and piperacillin in *C*. *jejuni* NCTC 11168 [130].

In addition to antimicrobial resistance genes, we examined the presence of heavy metal resistance genes, such as arsenic resistance genes. At high levels, arsenic is toxic to most cells, including microbial organisms, and is present in the natural environment [131, 132]. In this study, *arsP* was found in ST2084 *Campylobacter jejuni* and this gene has previously been associated with arsenic resistance [133]. Exposure to arsenic can select for resistant bacteria, including through horizontal gene transfer of arsenic resistance genes [134]; however, we did not collect any information about environmental levels of arsenic in areas in which samples were collected to assess whether the environmental arsenic exposure may have led to the acquisition of arsenic resistance genes by the studied *Campylobacter* isolates.

Previous studies have shown that different species of *Campylobacter* bacteria can form biofilms under laboratory conditions, including microaerophilic conditions [135]. However, in our study none of the tested *Campylobacter* isolates formed significantly more biofilm compared to the negative control. In comparison to monoculture, *C*. *jejuni* has previously been shown to produce more biofilm in a mixed culture containing *Pseudomonas aeruginosa* or *Escherichia coli* [136].

## Conclusions

The presence of *Campylobacter* in dairy products along the milk value chain in Ethiopia poses a potential risk for foodborne illness, especially due to the common practice of consuming raw or minimally processed cow milk in the country. The identification of identical *Campylobacter* isolates in both raw and pasteurized milk suggests transmission and underscores the need for improved hygiene and safety measures in the dairy value chain to improve control of *Campylobacter* and enhance the safety of dairy products. Lastly, contamination of both raw and pasteurized milk with fluoroquinolone-resistant strains highlights the need for effective control measures and antimicrobial stewardship.

## Supporting information

S1 File (XLSX)
